# Prevalence of Conduct Disorder in the Middle East: A Systematic Review and Meta-Analysis Protocol

**Published:** 2015-09

**Authors:** Maryam Salmanian, Mohammad Reza Mohammadi, Aabbas Ali Keshtkar, Fatemeh Asadian-koohestani, Seyyed Salman Alavi, Neda Sepasi

**Affiliations:** 1 Psychiatry and Psychology Research Center, Tehran University of Medical Sciences, Tehran, Iran; 2 Department of Epidemiology, Tehran University of Medical Sciences, Tehran, Iran

**Keywords:** *Conduct Disorder*, *the Middle East*, *Systematic Review*, *Prevalence*

## Abstract

**Background:** The global burden of conduct disorder is a major public health concern. Although there are different reports on the prevalence of conduct disorder in different Middle Eastern countries, to date, no research has reviewed them. Therefore, we aimed to conduct a systematic review and meta-analysis on the literature and present the prevalence of conduct disorder among children and adolescents in Middle Eastern countries.

**Methods:** Those cross-sectional studies with any type of random or non-random sampling, which described the prevalence of conduct disorder prior to age of 18, for at least one gender in the general or school-based populations who resided in Middle Eastern countries were included in this review. The scientific databases of PubMed, Scopus, Google Scholar, Index Medicus for the Eastern Mediterranean Region (IMEMR), Islamic World Science Citation Center (ISC), and Grey Literature including conference proceedings, and hand searching of key journals were searched from 1995 to the end of 2014. Two reviewers assessed the quality of the included studies independently and extracted the relevant data.

**Discussion:** This review provided a picture of different frequencies of conduct disorder in Middle Eastern countries and analyzed the sources of heterogeneity.

**Systematic Review Registration:** PROSPERO CRD42014014996

Conduct disorder is characterized by aggressive behaviors towards people and animals, deceitfulness or theft, destruction of property and serious violations of rules which persist in the child or adolescent during the past 12 months prior to age 18 (1).

The global burden of conduct disorder is a major public health concern, particularly in males (2). The worldwide prevalence of conduct disorder was reported to be 3.6% for males and 1.5% for females, which was consistent in three time periods of 1990, 2005 and 2010(3).

Risk factors of conduct disorder include individual, family and social factors. Individual factors consist of impulsiveness, low autonomic baseline arousal, reduced excitability particularly to punishing stimuli, accelerated habituation, poor verbal skills, impairment in executive functioning, low orienting reaction, low IQ and low educational achievement; family factors involve poor parental supervision, inconsistent, neglectful or harsh discipline, authoritarian style of parenting, child abuse, parental conflict and disrupted families, antisocial parents and large family size; and social factors include low socioeconomic level, delinquent peers, schools with high delinquency rate and high crime neighborhoods (4-7).

Several psychological interventions were suggested to reduce conduct disorder symptoms such as family and parenting interventions, multi-systemic therapy, parent management training, functional family therapy, various behavioral management strategies, multidimensional treatment foster care and cognitive-behavior skills building programs (8-10).

Several studies reported the prevalence of conduct disorder in different Middle Eastern countries; for instance, Mohammadi et al. (2014) revealed a prevalence rate of 32.9% for conduct disorder in Iranian children and adolescents (11). Another research which was conducted on 6–12-year-old children in Egypt reported the prevalence of conduct disorder to be 25.3% (12).

In this systematic review, we aimed to investigate the prevalence of conduct disorder in the Middle East. Although there are different reports of prevalence of conduct disorder in different regions of the Middle East, to date, no research has reviewed them. Therefore, we conducted a systematic review and meta-analysis on the literature and presented the prevalence of conduct disorder among children and adolescents in the Middle East. Also, the heterogeneity of conduct disorder frequencies in the various countries of the Middle East was assessed, and the potential sources of heterogeneity were analyzed.

## Methods

***Inclusion and Exclusion Criteria***

Cross-sectional studies including descriptive and survey studies, with any type of random or non-random sampling were included in this systematic review. The included studies described the prevalence of conduct disorder among children and adolescents prior to age of 18 for both genders, and at least one gender, in the general or school-based populations, residing in Middle Eastern countries. Moreover, the included studies defined conduct disorder based on the third, fourth and fifth versions of the Diagnostic and Statistical Manual of Mental Disorders (DSM).

We excluded clinical or interventional studies, and researches reporting the prevalence of conduct disorder with comorbidities, and we also excluded those studies conducted on single or high-risk groups.

***Search Strategy***

To identify the included studies, we systematically searched five databases including PubMed, Scopus, Google Scholar, Index Medicus for the Eastern Mediterranean Region (IMEMR), and Islamic World Science Citation Center (ISC). We also searched grey literature including conference proceedings. Moreover, hand searching of key journals and reference lists of all the included studies were reviewed. Moreover, we searched all studies from 1995 to the end of 2014. 

The following key-terms were included: Conduct disorder, prevalence, and The Middle Eastern countries (Table 1). Also, title and abstract tags were used for “conduct disorder”, and “prevalence” search terms. Each member of the “Middle Eastern countries” was searched by title and abstract, place of journal publication, and affiliation tags. Search terms were combined with “OR” and “AND” operators. 

The two authors selected the included studies independently based on the full texts.

**Data Collection and Analysis**

***Data Extraction***

Relevant data were independently extracted from the included studies and entered into the data extraction form by the two reviewers, who resolved the inconsistencies of the extracted data through consensus. The extracted data included the followings: The first author, publication year, journal, survey years, study design, name and population of the study location (country, province/state, city), district of study location (urban/rural), sample size, response rate, gender, age, type of sampling, sample population (general/school-based), type of school (state/private), instruments, paternal and maternal occupation, level of paternal and maternal education, number of siblings, family income, maternal and paternal loss, addiction and crime of family. 

***Quality Assessment***

For this review, we used the method and result sections of the STROBE checklist for quality assessment of the selected cross-sectional studies (13). This tool evaluates the quality of included studies across six core components including study design, participants, bias, measurement, descriptive and outcome data. This review was done according to PRISMA checklist. Two reviewers assessed the quality of included studies independently. 

***Statistical Analysis and Data Synthesis***

We used graphical methods and fixed or random effect models for the statistical analysis of the data. Sub-groups analysis or meta-regression was used to assess the sources of heterogeneity. Also, sensitivity analysis was performed, and publication bias was assessed using graphical methods and statistical tests. 

## Discussion

In this review, we investigated the studies conducted on the prevalence of conduct disorder in Middle Eastern countries from 1995 to the end of 2014 in order to analyze the sources of heterogeneity. The findings revealed a picture of different frequencies of conduct disorder in Middle Eastern countries, and contributed to a better understanding of the heterogeneities among the various reported prevalence rates of conduct disorder. The findings of this review may improve policy decisions, leading to a reduction in conduct disorder symptoms and providing improved special services for children and adolescents with conduct disorder.

## Limitation

We will not be able to combine or meta-analyze the results, if there is methodological heterogeneity between the studies. Also, we may not be able to access to all gray literature in our review.

## Conclusion

This review provided a picture of different frequencies of conduct disorder in Middle Eastern countries and analyzed the sources of heterogeneity.

**Table 1:** Search Terms of the Systematic Review in Prevalence of Conduct Disorder in Middle East

**Concept**	**Search Terms**
Conduct Disorder	*MeSH Term: Conduct disorder / Free-text term: Conduct problem*
Prevalence	MeSH terms: Prevalence, Incidence, epidemiology* / Free-text terms: rate, frequency
The Middle Eastern Countries	*MeSH Terms: Middle East, Near East, West Bank, Gaza Strip, Arab countries / MeSH terms for each Middle East Member Country: Afghanistan, Bahrain, Egypt, Iran, Iraq, Israel, Jordan, Kuwait, Lebanon, Oman, Qatar, Saudi Arabia, Syria, Turkey, United Arab Emirates, Yemen / Free-text terms for other Middle East Member Countries: Cyprus, Palestine*
